# Persistent Pulmonary Atelectasis After Tracheoesophageal Fistula Repair: Diagnostic Challenge of a Communicating Bronchopulmonary Foregut Malformation Group 1a in a Neonate With VACTERL Association

**DOI:** 10.1002/kjm2.70184

**Published:** 2026-03-02

**Authors:** Tien‐Pai Huang, Twei‐Shiun Jaw, Yu‐Tang Chang, Yu‐Han Chen

**Affiliations:** ^1^ Department of Radiology Kaohsiung Medical University Hospital Kaohsiung Taiwan; ^2^ Department of Radiology, School of Medicine College of Medicine, Kaohsiung Medical University Kaohsiung Taiwan; ^3^ Division of Pediatric Surgery, Department of Surgery Kaohsiung Medical University Hospital Kaohsiung Taiwan

Communicating bronchopulmonary foregut malformations (CBPFMs) are rare developmental abnormalities characterized by a congenital communication or fistula between an isolated portion of pulmonary tissue and the upper gastrointestinal tract [1]. The symptoms include neonatal respiratory distress, feeding difficulties, recurrent infections, and might be associated with esophageal atresia (EA). Early diagnosis and differentiation between tracheoesophageal fistula (TEF) and CBPFM‐combined EA is challenging; although, advantageously, these two distinct entities exhibit different characteristics: TEF typically involves a fistulous connection at the distal trachea or carina with esophagus [2], whereas CBPFM is characterized by an abnormal bronchus or respiratory tract directly connecting to the esophagus or stomach [1].

A preterm female infant, born at 32 + 3 weeks' gestation (birth weight 1625 g; Apgar scores of 6 and 8 at 1 and 5 min respectively), presented with respiratory distress, a dangling right thumb, and imperforate anus with a rectovestibular fistula. Type C TEF was suspected after unsuccessful nasogastric tube placement [2] while an initial chest–abdominal radiograph (“babygram”) showed an air‐filled proximal esophageal pouch in the lower cervical region (Figure 1a). She was admitted to the Neonatal Intensive Care Unit and, at 2 days of age, underwent esophageal reconstruction with primary anastomosis; a transverse colostomy was created 1 week later. Subsequently, she developed persistent right‐lung atelectasis where a postoperative chest radiograph showed collapsed right lung with air bronchograms and a radiolucent tract communicating with the esophagus (Figure 1b). An esophagogram confirmed a fistulous tract between the mid‐esophagus and the right bronchus (Figure 1c). Chest computed tomography (CT) showed collapsed right lung with an abnormal communication to the lower third of the esophagus (Figure 1d); accordingly, CBPFM Group1a was diagnosed (Figure 1e) [1], and a right pneumonectomy was undertaken for refractory infection and a nonfunctional hypoplastic right lung. Histopathology disclosed a broncho‐esophageal fistula with concomitant cytomegalovirus infection, while echocardiography demonstrated a patent ductus arteriosus (PDA), atrial septal defect (ASD) and type II ventricular septal defect (VSD), consistent with VACTERL association. In stable condition, the patient was transferred to another tertiary center for PDA/VSD repair, subsequently remaining stable on high‐flow nasal cannula oxygen and nasogastric feeding. At her 10‐month outpatient follow‐up visit, her body weight had gradually increased to 6.5 kg, which was still below the 3rd percentile.

**FIGURE 1:**
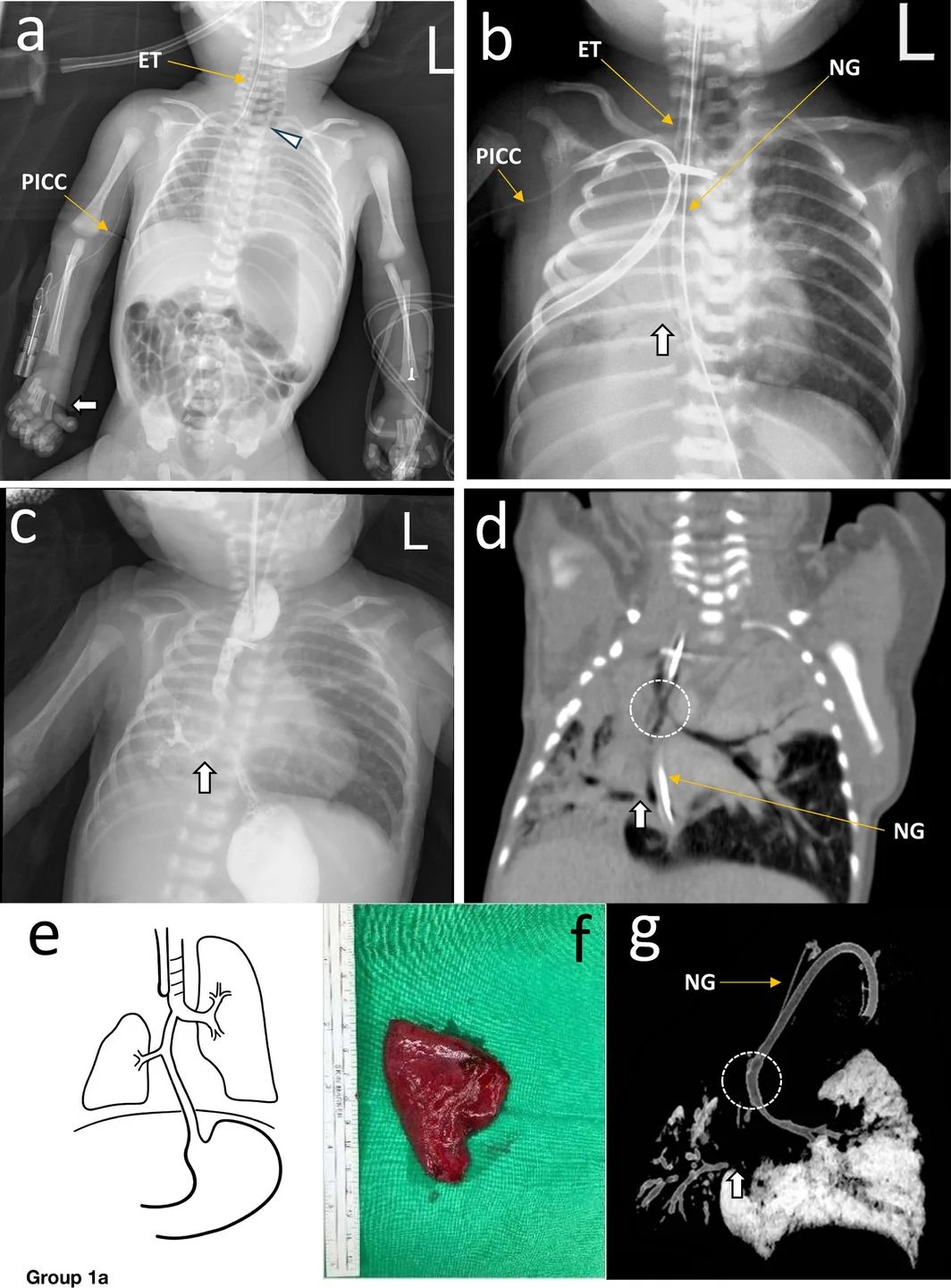
(a) Chest and abdomen babygram showed a radiolucent pouch at cervical region (arrowhead), absence of the right first metacarpal bone (arrow) with a hypoplastic right thumb, and a relatively smaller right lung. PICC: Peripherally inserted central catheter, ET: Endotracheal tube; (b) Chest radiograph obtained 2 days after TEF repair showed a well‐positioned nasogastric tube, right‐lung collapse with air bronchograms, and a radiolucent tract communicating with the esophagus (arrow). NG: Nasogastric tube; (c) Esophagography confirmed the abnormal connection between the right bronchus and the middle third of the esophagus. Metallic clips are also noted in the upper mediastinum, consistent with prior surgical repair of esophageal atresia; (d) Coronal CT shows absence of the right main bronchus (circle). The right lung is completely collapsed, and a broncho‐esophageal fistula (arrow) is connected to the esophagus; (e) Schematic illustration demonstrating Communicating Bronchopulmonary Foregut Malformation (CBPFM), Group 1a, according to the classification proposed by Srikanth et al. [1]. The illustrated anatomy is concordant with our case; (f) Gross surgical specimen showing a collapsed, hypoplastic right lung, status post resection; (g) Volume rendering image demonstrates absence of the right main bronchus (circle), associated non‐inflation of the right lung parenchyma, and a broncho‐esophageal fistula (arrow).

EA and TEF are often concomitant congenital anomalies, resulting in symptoms including the inability to pass food into the stomach and aspiration or choking. The prevalence of TEF varies from 1 to 4 in 10,000 births [2], with high association between TEF and VACTERAL association being widely accepted. According to a recent review, 22.2% of type C TEF cases were accompanied by VACTERL association; conversely, only 61 CBPFM cases were reported in major medical databases (PubMed, Ovid, EMBASE) from January 1992 to August 2018 [3]. The organizing spectrum of CBPFM was classified by Srikanth et al. in 1992 according to the association of esophageal atresia and normal ipsilateral bronchovascular system being existent or not [1]. This patient was consistent with CBPFM Group 1a, in which the entire hypoplastic lung—lacking an ipsilateral main bronchus—communicates with the esophagus. Because type C TEF is far more common than CBPFM, the latter is therefore easily initially misdiagnosed as TEF, particularly when EA is present [4]. A practical improvement would be earlier chest CT or bronchoscopy when ipsilateral lung hypoplasia is noted to facilitate earlier recognition of CBPFM.

## Conflicts of Interest

The authors declare no conflicts of interest.

## Data Availability

The data that support the findings of this study are available on request from the corresponding author. The data are not publicly available due to privacy or ethical restrictions.
